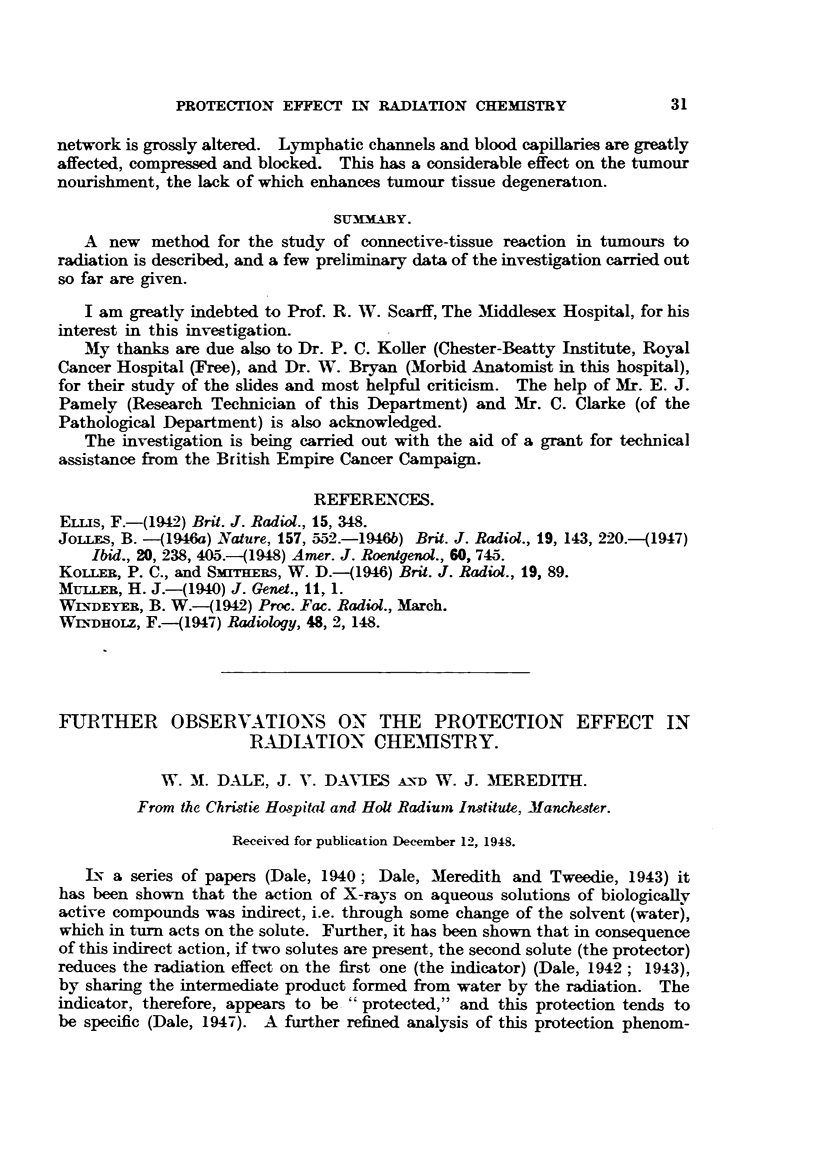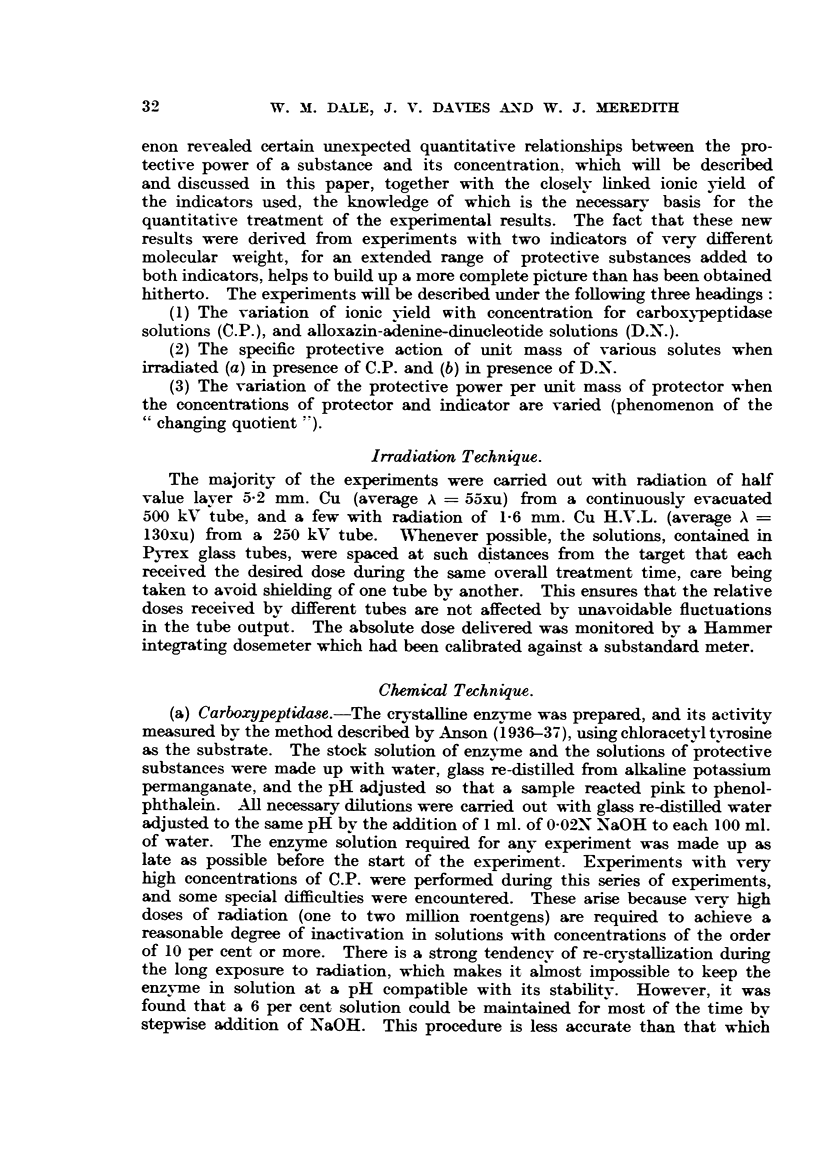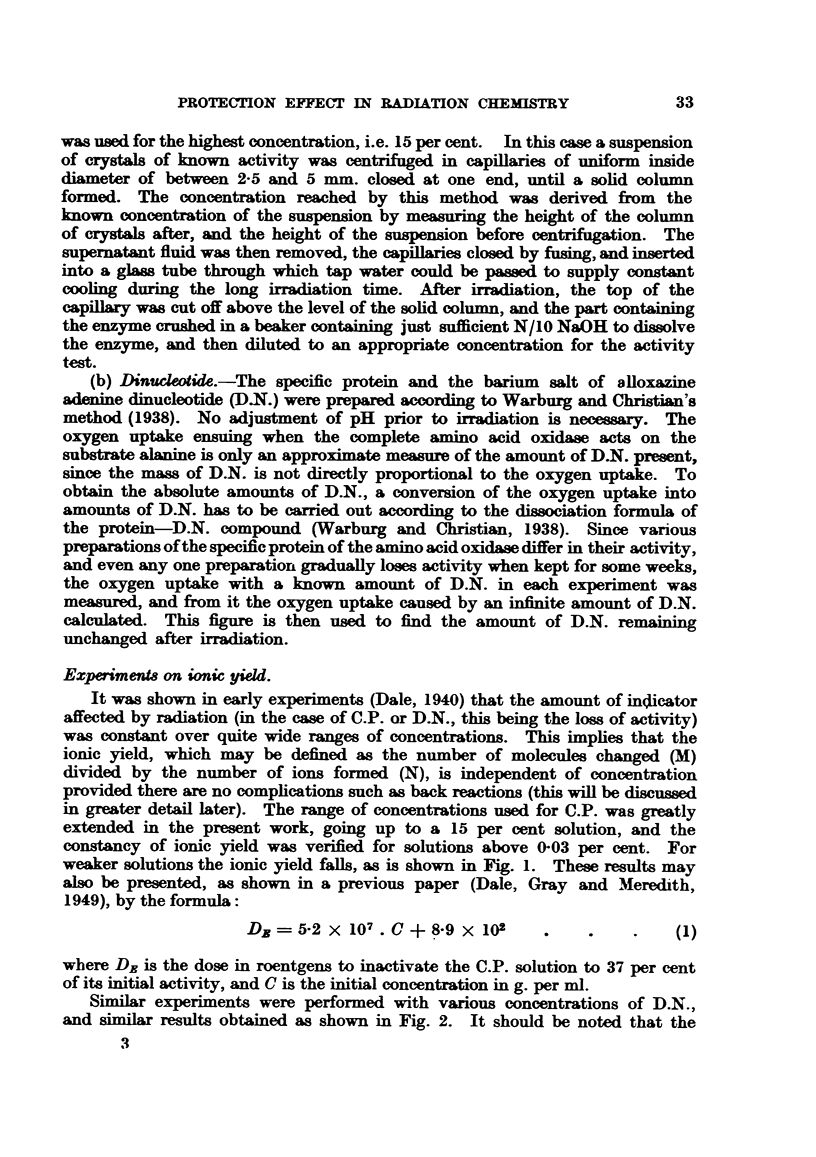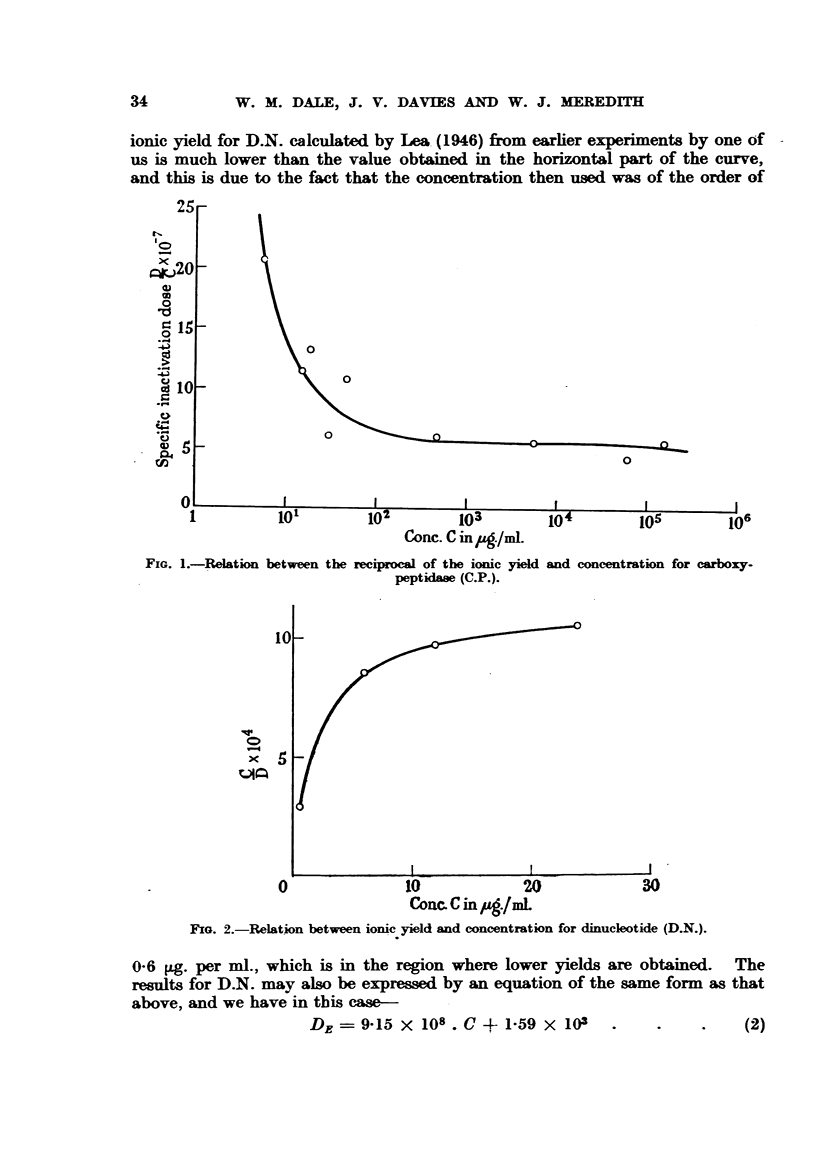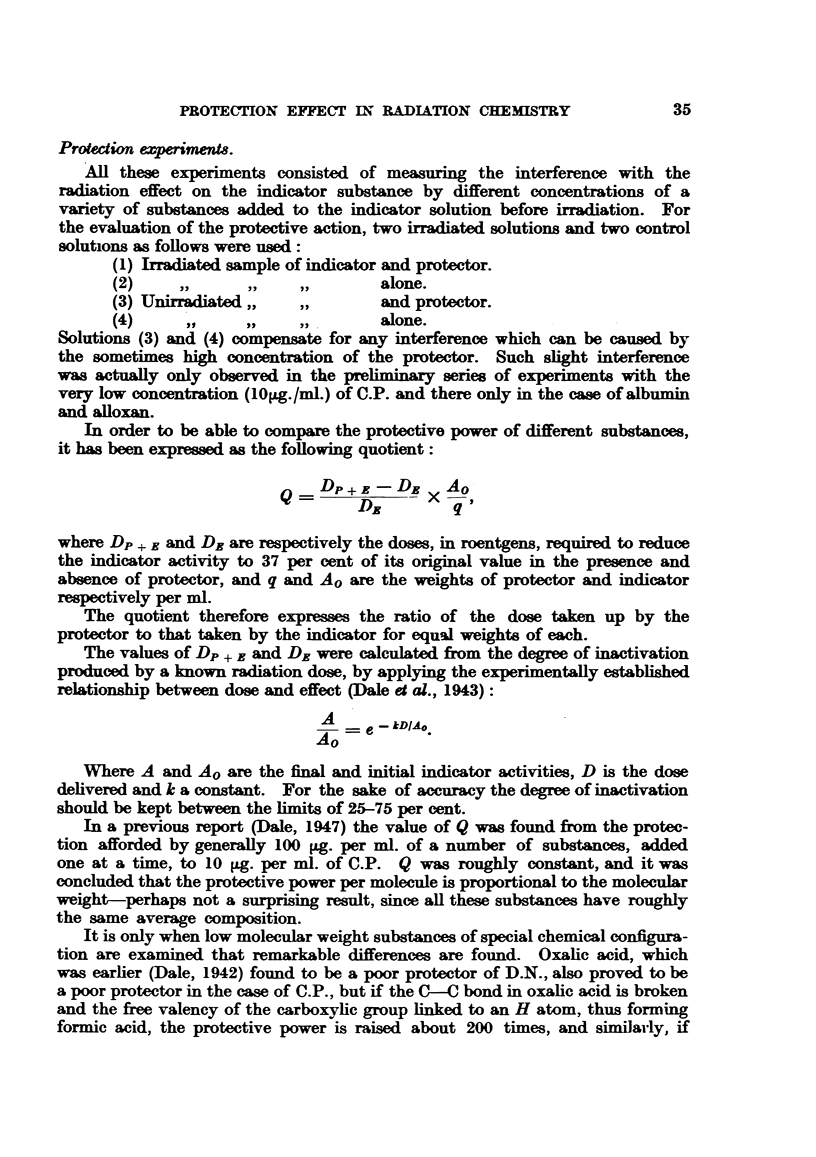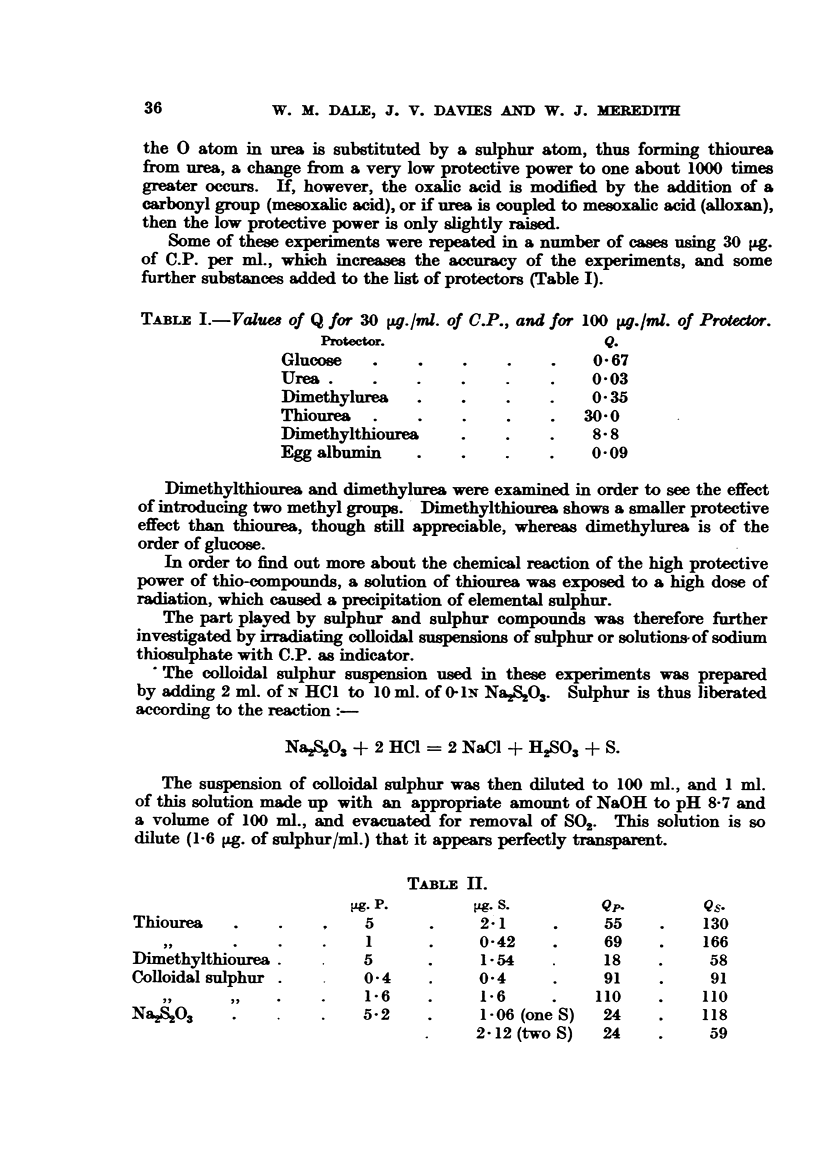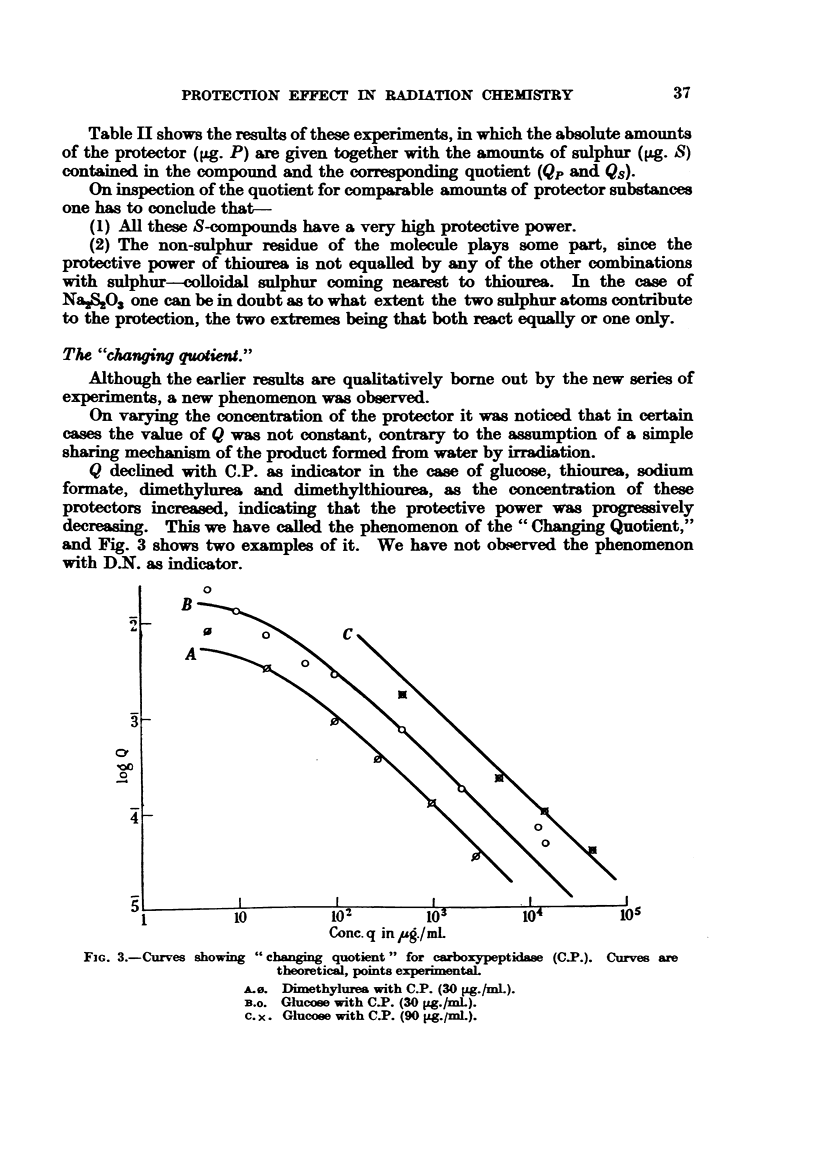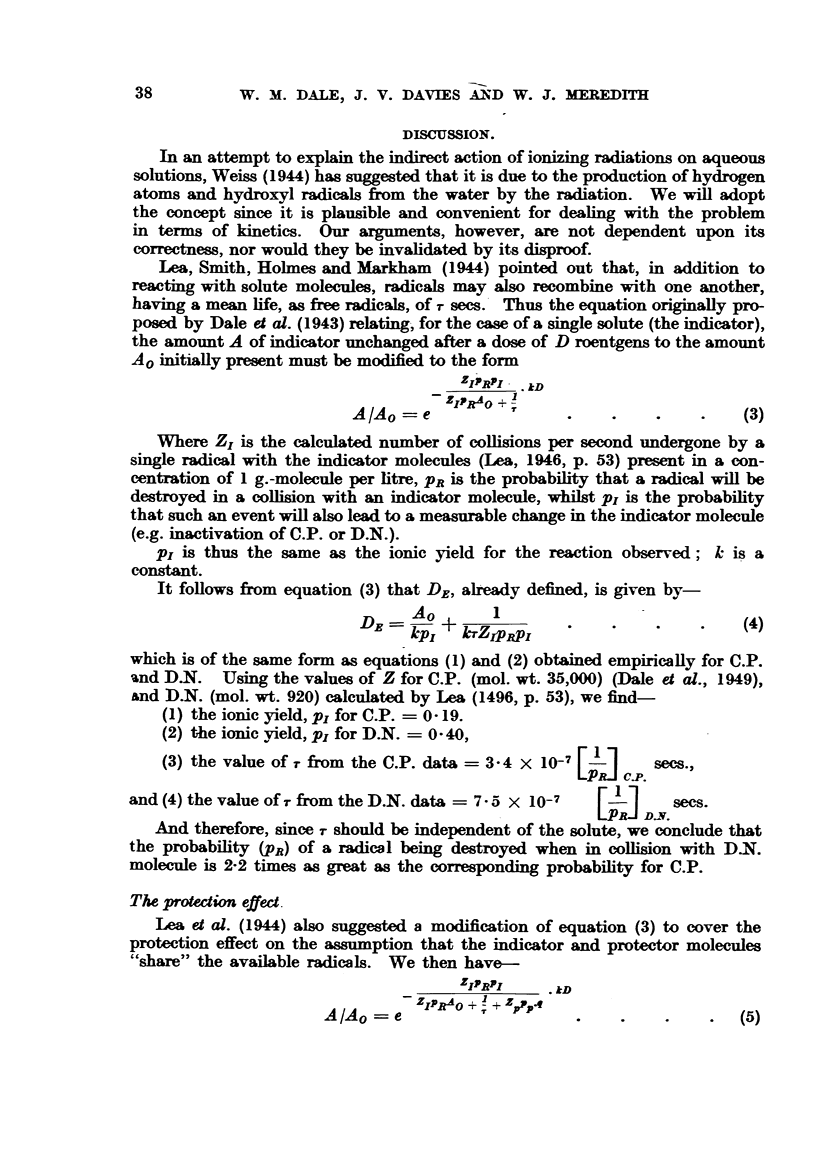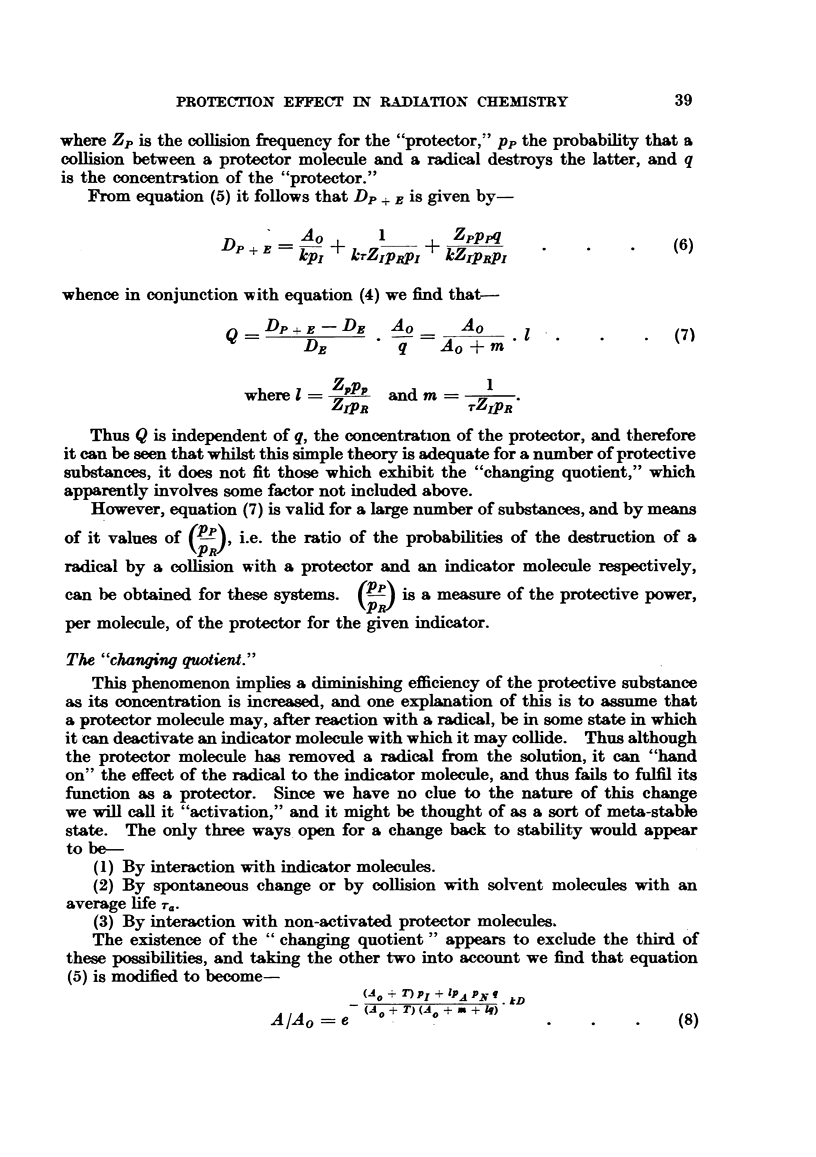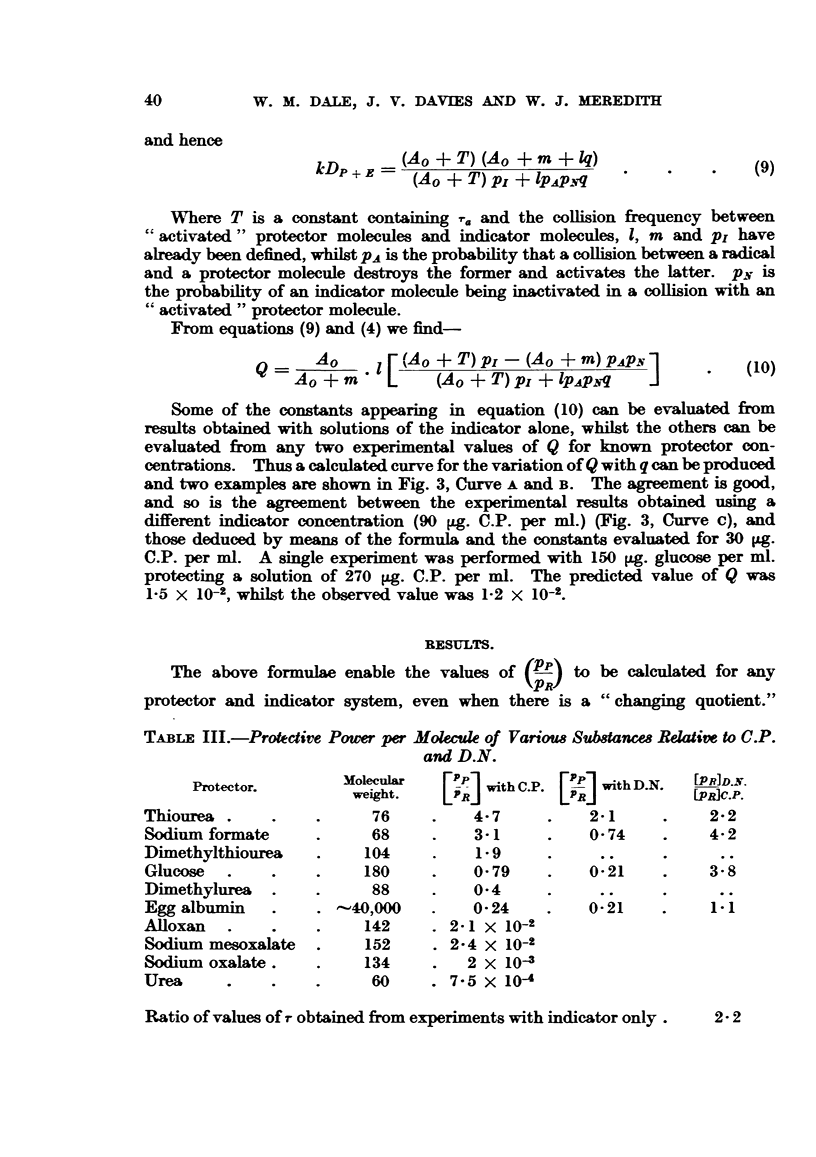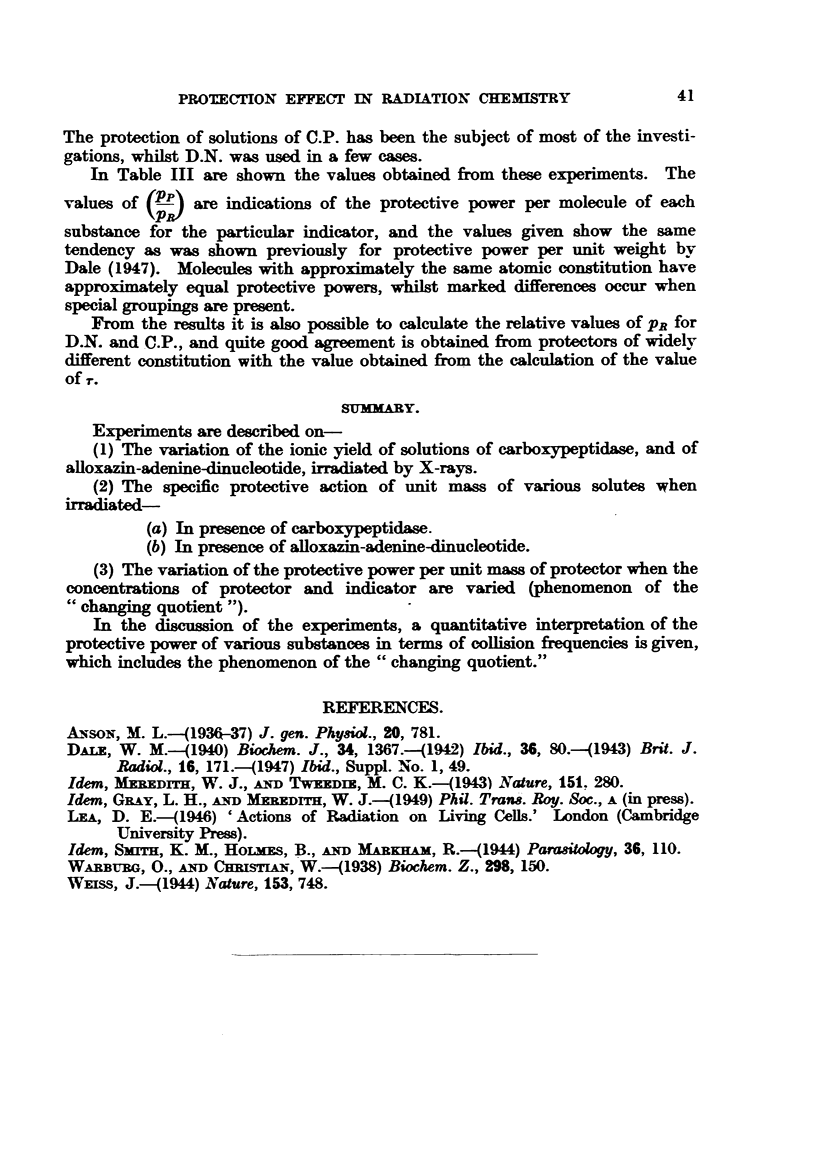# Further Observations on the Protection Effect in Radiation Chemistry

**DOI:** 10.1038/bjc.1949.4

**Published:** 1949-03

**Authors:** W. M. Dale, J. V. Davies, W. J. Meredith


REFERENCES.

ELLIS, F.—(1942) *Brit. J. Radiol.*, **15**, 348.
JOLLES, B. —(1946*a*) *Nature*, **157**, 552.—1946*b*) *Brit. J. Radiol.*, **19**, 143, 220.—(1947) *Ibid.*, **20**, 238, 405.—(1948) *Amer. J. Roentgenol.*, **60**, 745.
KOLLER, P. C., and SMITHERS, W. D.—(1946) *Brit. J. Radiol.*, **19**, 89.
MULLER, H. J.—(1940) *J. Genet.*, **11**, 1.
WINDEYER, B. W.—(1942) *Proc. Fac. Radiol.*, March.
WINDHOLZ, F.—(1947) *Radiology*, **48**, 2, 148.

### FURTHER OBSERVATIONS ON THE PROTECTION EFFECT IN RADIATION CHEMISTRY.

W. M. DALE, J. V. DAVIES AND W. J. MEREDITH.

*From the Christie Hospital and Holt Radium Institute, Manchester.*

Received for publication December 12, 1948.

IN a series of papers (Dale, 1940; Dale, Meredith and Tweedie, 1943) it has been shown that the action of X-rays on aqueous solutions of biologically active compounds was indirect, i.e. through some change of the solvent (water), which in turn acts on the solute. Further, it has been shown that in consequence of this indirect action, if two solutes are present, the second solute (the protector) reduces the radiation effect on the first one (the indicator) (Dale, 1942; 1943), by sharing the intermediate product formed from water by the radiation. The indicator, therefore, appears to be "protected," and this protection tends to be specific (Dale, 1947). A further refined analysis of this protection phenomenon revealed certain unexpected quantitative relationships between the protective power of a substance and its concentration, which will be described and discussed in this paper, together with the closely linked ionic yield of the indicators used, the knowledge of which is the necessary basis for the quantitative treatment of the experimental results. The fact that these new results were derived from experiments with two indicators of very different molecular weight, for an extended range of protective substances added to both indicators, helps to build up a more complete picture than has been obtained hitherto. The experiments will be described under the following three headings :

(1) The variation of ionic yield with concentration for carboxypeptidase solutions (C.P.), and alloxazin-adenine-dinucleotide solutions (D.N.).

(2) The specific protective action of unit mass of various solutes when irradiated (*a*) in presence of C.P. and (*b*) in presence of D.N.

(3) The variation of the protective power per unit mass of protector when the concentrations of protector and indicator are varied (phenomenon of the " changing quotient ").

*Irradiation Technique.*

The majority of the experiments were carried out with radiation of half value layer 5·2 mm. Cu (average $\lambda = 55$xu) from a continuously evacuated 500 kV tube, and a few with radiation of 1·6 mm. Cu H.V.L. (average $\lambda = 130$xu) from a 250 kV tube. Whenever possible, the solutions, contained in Pyrex glass tubes, were spaced at such distances from the target that each received the desired dose during the same overall treatment time, care being taken to avoid shielding of one tube by another. This ensures that the relative doses received by different tubes are not affected by unavoidable fluctuations in the tube output. The absolute dose delivered was monitored by a Hammer integrating dosemeter which had been calibrated against a substandard meter.

*Chemical Technique.*

(a) *Carboxypeptidase.*—The crystalline enzyme was prepared, and its activity measured by the method described by Anson (1936–37), using chloracetyl tyrosine as the substrate. The stock solution of enzyme and the solutions of protective substances were made up with water, glass re-distilled from alkaline potassium permanganate, and the pH adjusted so that a sample reacted pink to phenolphthalein. All necessary dilutions were carried out with glass re-distilled water adjusted to the same pH by the addition of 1 ml. of 0·02N NaOH to each 100 ml. of water. The enzyme solution required for any experiment was made up as late as possible before the start of the experiment. Experiments with very high concentrations of C.P. were performed during this series of experiments, and some special difficulties were encountered. These arise because very high doses of radiation (one to two million roentgens) are required to achieve a reasonable degree of inactivation in solutions with concentrations of the order of 10 per cent or more. There is a strong tendency of re-crystallization during the long exposure to radiation, which makes it almost impossible to keep the enzyme in solution at a pH compatible with its stability. However, it was found that a 6 per cent solution could be maintained for most of the time by stepwise addition of NaOH. This procedure is less accurate than that which was used for the highest concentration, i.e. 15 per cent. In this case a suspension of crystals of known activity was centrifuged in capillaries of uniform inside diameter of between 2·5 and 5 mm. closed at one end, until a solid column formed. The concentration reached by this method was derived from the known concentration of the suspension by measuring the height of the column of crystals after, and the height of the suspension before centrifugation. The supernatant fluid was then removed, the capillaries closed by fusing, and inserted into a glass tube through which tap water could be passed to supply constant cooling during the long irradiation time. After irradiation, the top of the capillary was cut off above the level of the solid column, and the part containing the enzyme crushed in a beaker containing just sufficient N/10 NaOH to dissolve the enzyme, and then diluted to an appropriate concentration for the activity test.

(b) *Dinucleotide.*—The specific protein and the barium salt of alloxazine adenine dinucleotide (D.N.) were prepared according to Warburg and Christian's method (1938). No adjustment of pH prior to irradiation is necessary. The oxygen uptake ensuing when the complete amino acid oxidase acts on the substrate alanine is only an approximate measure of the amount of D.N. present, since the mass of D.N. is not directly proportional to the oxygen uptake. To obtain the absolute amounts of D.N., a conversion of the oxygen uptake into amounts of D.N. has to be carried out according to the dissociation formula of the protein—D.N. compound (Warburg and Christian, 1938). Since various preparations of the specific protein of the amino acid oxidase differ in their activity, and even any one preparation gradually loses activity when kept for some weeks, the oxygen uptake with a known amount of D.N. in each experiment was measured, and from it the oxygen uptake caused by an infinite amount of D.N. calculated. This figure is then used to find the amount of D.N. remaining unchanged after irradiation.

*Experiments on ionic yield.*

It was shown in early experiments (Dale, 1940) that the amount of indicator affected by radiation (in the case of C.P. or D.N., this being the loss of activity) was constant over quite wide ranges of concentrations. This implies that the ionic yield, which may be defined as the number of molecules changed (M) divided by the number of ions formed (N), is independent of concentration provided there are no complications such as back reactions (this will be discussed in greater detail later). The range of concentrations used for C.P. was greatly extended in the present work, going up to a 15 per cent solution, and the constancy of ionic yield was verified for solutions above 0·03 per cent. For weaker solutions the ionic yield falls, as is shown in Fig. 1. These results may also be presented, as shown in a previous paper (Dale, Gray and Meredith, 1949), by the formula:

$$D_E = 5{\cdot}2 \times 10^7 \, . \, C + 8{\cdot}9 \times 10^2 \quad . \quad . \quad . \quad (1)$$

where $D_E$ is the dose in roentgens to inactivate the C.P. solution to 37 per cent of its initial activity, and $C$ is the initial concentration in g. per ml.

Similar experiments were performed with various concentrations of D.N., and similar results obtained as shown in Fig. 2. It should be noted that the ionic yield for D.N. calculated by Lea (1946) from earlier experiments by one of us is much lower than the value obtained in the horizontal part of the curve, and this is due to the fact that the concentration then used was of the order of

FIG. 1.—Relation between the reciprocal of the ionic yield and concentration for carboxypeptidase (C.P.).

FIG. 2.—Relation between ionic yield and concentration for dinucleotide (D.N.).

0·6 μg. per ml., which is in the region where lower yields are obtained. The results for D.N. may also be expressed by an equation of the same form as that above, and we have in this case—

$$D_E = 9{\cdot}15 \times 10^8 \, . \, C + 1{\cdot}59 \times 10^3 \quad . \quad . \quad . \quad (2)$$

*Protection experiments.*

All these experiments consisted of measuring the interference with the radiation effect on the indicator substance by different concentrations of a variety of substances added to the indicator solution before irradiation. For the evaluation of the protective action, two irradiated solutions and two control solutions as follows were used:

(1) Irradiated sample of indicator and protector.
(2) ,, ,, ,, alone.
(3) Unirradiated ,, ,, and protector.
(4) ,, ,, ,, alone.

Solutions (3) and (4) compensate for any interference which can be caused by the sometimes high concentration of the protector. Such slight interference was actually only observed in the preliminary series of experiments with the very low concentration (10μg./ml.) of C.P. and there only in the case of albumin and alloxan.

In order to be able to compare the protective power of different substances, it has been expressed as the following quotient:

$$Q = \frac{D_{P+E} - D_E}{D_E} \times \frac{A_O}{q},$$

where $D_{P+E}$ and $D_E$ are respectively the doses, in roentgens, required to reduce the indicator activity to 37 per cent of its original value in the presence and absence of protector, and $q$ and $A_O$ are the weights of protector and indicator respectively per ml.

The quotient therefore expresses the ratio of the dose taken up by the protector to that taken by the indicator for equal weights of each.

The values of $D_{P+E}$ and $D_E$ were calculated from the degree of inactivation produced by a known radiation dose, by applying the experimentally established relationship between dose and effect (Dale *et al.*, 1943):

$$\frac{A}{A_O} = e^{-kD/A_O}.$$

Where $A$ and $A_O$ are the final and initial indicator activities, $D$ is the dose delivered and $k$ a constant. For the sake of accuracy the degree of inactivation should be kept between the limits of 25–75 per cent.

In a previous report (Dale, 1947) the value of $Q$ was found from the protection afforded by generally 100 μg. per ml. of a number of substances, added one at a time, to 10 μg. per ml. of C.P. $Q$ was roughly constant, and it was concluded that the protective power per molecule is proportional to the molecular weight—perhaps not a surprising result, since all these substances have roughly the same average composition.

It is only when low molecular weight substances of special chemical configuration are examined that remarkable differences are found. Oxalic acid, which was earlier (Dale, 1942) found to be a poor protector of D.N., also proved to be a poor protector in the case of C.P., but if the C—C bond in oxalic acid is broken and the free valency of the carboxylic group linked to an $H$ atom, thus forming formic acid, the protective power is raised about 200 times, and similarly, if the O atom in urea is substituted by a sulphur atom, thus forming thiourea from urea, a change from a very low protective power to one about 1000 times greater occurs. If, however, the oxalic acid is modified by the addition of a carbonyl group (mesoxalic acid), or if urea is coupled to mesoxalic acid (alloxan), then the low protective power is only slightly raised.

Some of these experiments were repeated in a number of cases using 30 μg. of C.P. per ml., which increases the accuracy of the experiments, and some further substances added to the list of protectors (Table I).

TABLE I.—*Values of Q for 30 μg./ml. of C.P., and for 100 μg./ml. of Protector.*

| Protector. | *Q.* |
|---|---|
| Glucose | 0·67 |
| Urea | 0·03 |
| Dimethylurea | 0·35 |
| Thiourea | 30·0 |
| Dimethylthiourea | 8·8 |
| Egg albumin | 0·09 |

Dimethylthiourea and dimethylurea were examined in order to see the effect of introducing two methyl groups. Dimethylthiourea shows a smaller protective effect than thiourea, though still appreciable, whereas dimethylurea is of the order of glucose.

In order to find out more about the chemical reaction of the high protective power of thio-compounds, a solution of thiourea was exposed to a high dose of radiation, which caused a precipitation of elemental sulphur.

The part played by sulphur and sulphur compounds was therefore further investigated by irradiating colloidal suspensions of sulphur or solutions of sodium thiosulphate with C.P. as indicator.

The colloidal sulphur suspension used in these experiments was prepared by adding 2 ml. of N HCl to 10 ml. of 0·1N $Na_2S_2O_3$. Sulphur is thus liberated according to the reaction :—

$$Na_2S_2O_3 + 2\ HCl = 2\ NaCl + H_2SO_3 + S.$$

The suspension of colloidal sulphur was then diluted to 100 ml., and 1 ml. of this solution made up with an appropriate amount of NaOH to pH 8·7 and a volume of 100 ml., and evacuated for removal of $SO_2$. This solution is so dilute (1·6 μg. of sulphur/ml.) that it appears perfectly transparent.

TABLE II.

| | μg. P. | μg. S. | $Q_P$. | $Q_S$. |
|---|---|---|---|---|
| Thiourea | 5 | 2·1 | 55 | 130 |
| ,, | 1 | 0·42 | 69 | 166 |
| Dimethylthiourea | 5 | 1·54 | 18 | 58 |
| Colloidal sulphur | 0·4 | 0·4 | 91 | 91 |
| ,, ,, | 1·6 | 1·6 | 110 | 110 |
| $Na_2S_2O_3$ | 5·2 | 1·06 (one S) | 24 | 118 |
| | | 2·12 (two S) | 24 | 59 |

Table II shows the results of these experiments, in which the absolute amounts of the protector (μg. $P$) are given together with the amounts of sulphur (μg. $S$) contained in the compound and the corresponding quotient ($Q_P$ and $Q_S$).

On inspection of the quotient for comparable amounts of protector substances one has to conclude that—

(1) All these $S$-compounds have a very high protective power.

(2) The non-sulphur residue of the molecule plays some part, since the protective power of thiourea is not equalled by any of the other combinations with sulphur—colloidal sulphur coming nearest to thiourea. In the case of $Na_2S_2O_3$ one can be in doubt as to what extent the two sulphur atoms contribute to the protection, the two extremes being that both react equally or one only.

*The "changing quotient."*

Although the earlier results are qualitatively borne out by the new series of experiments, a new phenomenon was observed.

On varying the concentration of the protector it was noticed that in certain cases the value of $Q$ was not constant, contrary to the assumption of a simple sharing mechanism of the product formed from water by irradiation.

$Q$ declined with C.P. as indicator in the case of glucose, thiourea, sodium formate, dimethylurea and dimethylthiourea, as the concentration of these protectors increased, indicating that the protective power was progressively decreasing. This we have called the phenomenon of the "Changing Quotient," and Fig. 3 shows two examples of it. We have not observed the phenomenon with D.N. as indicator.

FIG. 3.—Curves showing "changing quotient" for carboxypeptidase (C.P.). Curves are theoretical, points experimental.

A. ø. Dimethylurea with C.P. (30 μg./ml.).

B. o. Glucose with C.P. (30 μg./ml.).

C. ×. Glucose with C.P. (90 μg./ml.).

DISCUSSION.

In an attempt to explain the indirect action of ionizing radiations on aqueous solutions, Weiss (1944) has suggested that it is due to the production of hydrogen atoms and hydroxyl radicals from the water by the radiation. We will adopt the concept since it is plausible and convenient for dealing with the problem in terms of kinetics. Our arguments, however, are not dependent upon its correctness, nor would they be invalidated by its disproof.

Lea, Smith, Holmes and Markham (1944) pointed out that, in addition to reacting with solute molecules, radicals may also recombine with one another, having a mean life, as free radicals, of $\tau$ secs. Thus the equation originally proposed by Dale *et al.* (1943) relating, for the case of a single solute (the indicator), the amount $A$ of indicator unchanged after a dose of $D$ roentgens to the amount $A_O$ initially present must be modified to the form

$$A/A_O = e^{-\frac{z_I p_R p_I}{z_I p_R A_O + \frac{1}{\tau}} \cdot kD} \quad . \quad . \quad . \quad . \quad . \quad (3)$$

Where $Z_I$ is the calculated number of collisions per second undergone by a single radical with the indicator molecules (Lea, 1946, p. 53) present in a concentration of 1 g.-molecule per litre, $p_R$ is the probability that a radical will be destroyed in a collision with an indicator molecule, whilst $p_I$ is the probability that such an event will also lead to a measurable change in the indicator molecule (e.g. inactivation of C.P. or D.N.).

$p_I$ is thus the same as the ionic yield for the reaction observed; $k$ is a constant.

It follows from equation (3) that $D_E$, already defined, is given by—

$$D_E = \frac{A_O}{kp_I} + \frac{1}{k\tau Z_I p_R p_I} \quad . \quad . \quad . \quad . \quad . \quad (4)$$

which is of the same form as equations (1) and (2) obtained empirically for C.P. and D.N. Using the values of $Z$ for C.P. (mol. wt. 35,000) (Dale *et al.*, 1949), and D.N. (mol. wt. 920) calculated by Lea (1496, p. 53), we find—

(1) the ionic yield, $p_I$ for C.P. $= 0\cdot19$.

(2) the ionic yield, $p_I$ for D.N. $= 0\cdot40$,

(3) the value of $\tau$ from the C.P. data $= 3\cdot4 \times 10^{-7} \left[\frac{1}{p_R}\right]_{C.P.}$ secs., and (4) the value of $\tau$ from the D.N. data $= 7\cdot5 \times 10^{-7} \left[\frac{1}{p_R}\right]_{D.N.}$ secs.

And therefore, since $\tau$ should be independent of the solute, we conclude that the probability ($p_R$) of a radical being destroyed when in collision with D.N. molecule is 2·2 times as great as the corresponding probability for C.P.

*The protection effect.*

Lea *et al.* (1944) also suggested a modification of equation (3) to cover the protection effect on the assumption that the indicator and protector molecules "share" the available radicals. We then have—

$$A/A_O = e^{-\frac{z_I p_R p_I}{z_I p_R A_O + \frac{1}{\tau} + z_p p_p q} \cdot kD} \quad . \quad . \quad . \quad . \quad (5)$$

where $Z_P$ is the collision frequency for the "protector," $p_P$ the probability that a collision between a protector molecule and a radical destroys the latter, and $q$ is the concentration of the "protector."

From equation (5) it follows that $D_{P+E}$ is given by—

$$D_{P+E} = \frac{A_O}{kp_I} + \frac{1}{k\tau Z_I p_R p_I} + \frac{Z_P p_P q}{kZ_I p_R p_I} \quad . \quad . \quad . \quad (6)$$

whence in conjunction with equation (4) we find that—

$$Q = \frac{D_{P+E} - D_E}{D_E} \cdot \frac{A_O}{q} = \frac{A_O}{A_O + m} \cdot l \quad . \quad . \quad . \quad (7)$$

$$\text{where } l = \frac{Z_p p_p}{Z_I p_R} \quad \text{and } m = \frac{1}{\tau Z_I p_R}.$$

Thus $Q$ is independent of $q$, the concentration of the protector, and therefore it can be seen that whilst this simple theory is adequate for a number of protective substances, it does not fit those which exhibit the "changing quotient," which apparently involves some factor not included above.

However, equation (7) is valid for a large number of substances, and by means of it values of $\left(\frac{p_P}{p_R}\right)$, i.e. the ratio of the probabilities of the destruction of a radical by a collision with a protector and an indicator molecule respectively, can be obtained for these systems. $\left(\frac{p_P}{p_R}\right)$ is a measure of the protective power, per molecule, of the protector for the given indicator.

*The "changing quotient."*

This phenomenon implies a diminishing efficiency of the protective substance as its concentration is increased, and one explanation of this is to assume that a protector molecule may, after reaction with a radical, be in some state in which it can deactivate an indicator molecule with which it may collide. Thus although the protector molecule has removed a radical from the solution, it can "hand on" the effect of the radical to the indicator molecule, and thus fails to fulfil its function as a protector. Since we have no clue to the nature of this change we will call it "activation," and it might be thought of as a sort of meta-stable state. The only three ways open for a change back to stability would appear to be—

(1) By interaction with indicator molecules.

(2) By spontaneous change or by collision with solvent molecules with an average life $\tau_a$.

(3) By interaction with non-activated protector molecules.

The existence of the " changing quotient " appears to exclude the third of these possibilities, and taking the other two into account we find that equation (5) is modified to become—

$$A/A_O = e^{-\frac{(A_0 + T)\, p_I + l p_A p_N q}{(A_0 + T)(A_0 + m + lq)} \cdot kD} \quad . \quad . \quad . \quad (8)$$

and hence

$$kD_{P+E} = \frac{(A_O + T)(A_O + m + lq)}{(A_O + T)\,p_I + lp_A p_N q} \quad . \quad . \quad . \quad (9)$$

Where $T$ is a constant containing $\tau_a$ and the collision frequency between "activated" protector molecules and indicator molecules, $l$, $m$ and $p_I$ have already been defined, whilst $p_A$ is the probability that a collision between a radical and a protector molecule destroys the former and activates the latter. $p_N$ is the probability of an indicator molecule being inactivated in a collision with an "activated" protector molecule.

From equations (9) and (4) we find—

$$Q = \frac{A_O}{A_O + m} \cdot l\left[\frac{(A_O + T)\,p_I - (A_O + m)\,p_A p_N}{(A_O + T)\,p_I + lp_A p_N q}\right] \quad . \quad (10)$$

Some of the constants appearing in equation (10) can be evaluated from results obtained with solutions of the indicator alone, whilst the others can be evaluated from any two experimental values of $Q$ for known protector concentrations. Thus a calculated curve for the variation of $Q$ with $q$ can be produced and two examples are shown in Fig. 3, Curve A and B. The agreement is good, and so is the agreement between the experimental results obtained using a different indicator concentration (90 μg. C.P. per ml.) (Fig. 3, Curve C), and those deduced by means of the formula and the constants evaluated for 30 μg. C.P. per ml. A single experiment was performed with 150 μg. glucose per ml. protecting a solution of 270 μg. C.P. per ml. The predicted value of $Q$ was $1{\cdot}5 \times 10^{-2}$, whilst the observed value was $1{\cdot}2 \times 10^{-2}$.

### RESULTS.

The above formulae enable the values of $\left(\frac{p_P}{p_R}\right)$ to be calculated for any protector and indicator system, even when there is a "changing quotient."

TABLE III.—*Protective Power per Molecule of Various Substances Relative to C.P. and D.N.*

| Protector. | Molecular weight. | $\left[\frac{p_P}{p_R}\right]$ with C.P. | $\left[\frac{p_P}{p_R}\right]$ with D.N. | $\frac{[p_R]D.N.}{[p_R]C.P.}$ |
|---|---|---|---|---|
| Thiourea | 76 | 4·7 | 2·1 | 2·2 |
| Sodium formate | 68 | 3·1 | 0·74 | 4·2 |
| Dimethylthiourea | 104 | 1·9 | .. | .. |
| Glucose | 180 | 0·79 | 0·21 | 3·8 |
| Dimethylurea | 88 | 0·4 | .. | .. |
| Egg albumin | ~40,000 | 0·24 | 0·21 | 1·1 |
| Alloxan | 142 | $2{\cdot}1 \times 10^{-2}$ | | |
| Sodium mesoxalate | 152 | $2{\cdot}4 \times 10^{-2}$ | | |
| Sodium oxalate | 134 | $2 \times 10^{-3}$ | | |
| Urea | 60 | $7{\cdot}5 \times 10^{-4}$ | | |

Ratio of values of $\tau$ obtained from experiments with indicator only . 2·2

The protection of solutions of C.P. has been the subject of most of the investigations, whilst D.N. was used in a few cases.

In Table III are shown the values obtained from these experiments. The values of $\left(\frac{p_P}{p_R}\right)$ are indications of the protective power per molecule of each substance for the particular indicator, and the values given show the same tendency as was shown previously for protective power per unit weight by Dale (1947). Molecules with approximately the same atomic constitution have approximately equal protective powers, whilst marked differences occur when special groupings are present.

From the results it is also possible to calculate the relative values of $p_R$ for D.N. and C.P., and quite good agreement is obtained from protectors of widely different constitution with the value obtained from the calculation of the value of $\tau$.

### SUMMARY.

Experiments are described on—

(1) The variation of the ionic yield of solutions of carboxypeptidase, and of alloxazin-adenine-dinucleotide, irradiated by X-rays.

(2) The specific protective action of unit mass of various solutes when irradiated—

(*a*) In presence of carboxypeptidase.

(*b*) In presence of alloxazin-adenine-dinucleotide.

(3) The variation of the protective power per unit mass of protector when the concentrations of protector and indicator are varied (phenomenon of the "changing quotient").

In the discussion of the experiments, a quantitative interpretation of the protective power of various substances in terms of collision frequencies is given, which includes the phenomenon of the "changing quotient."

### REFERENCES.

Anson, M. L.—(1936–37) *J. gen. Physiol.*, **20**, 781.

Dale, W. M.—(1940) *Biochem. J.*, **34**, 1367.—(1942) *Ibid.*, **36**, 80.—(1943) *Brit. J. Radiol.*, **16**, 171.—(1947) *Ibid.*, Suppl. No. 1, 49.

*Idem*, Meredith, W. J., and Tweedie, M. C. K.—(1943) *Nature*, **151**, 280.

*Idem*, Gray, L. H., and Meredith, W. J.—(1949) *Phil. Trans. Roy. Soc.*, A (in press).

Lea, D. E.—(1946) 'Actions of Radiation on Living Cells.' London (Cambridge University Press).

*Idem*, Smith, K. M., Holmes, B., and Markham, R.—(1944) *Parasitology*, **36**, 110.

Warburg, O., and Christian, W.—(1938) *Biochem. Z.*, **298**, 150.

Weiss, J.—(1944) *Nature*, **153**, 748.